# MKP-1 reduces Aβ generation and alleviates cognitive impairments in Alzheimer’s disease models

**DOI:** 10.1038/s41392-019-0091-4

**Published:** 2019-12-06

**Authors:** Yehong Du, Yexiang Du, Yun Zhang, Zhilin Huang, Min Fu, Junjie Li, Yayan Pang, Peng Lei, Yu Tian Wang, Weihong Song, Guiqiong He, Zhifang Dong

**Affiliations:** 1 Pediatric Research Institute, Ministry of Education Key Laboratory of Child Development and Disorders, National Clinical Research Center for Child Health and Disorders, China International Science and Technology Cooperation Base of Child Development and Critical Disorders, Chongqing Key Laboratory of Translational Medical Research in Cognitive Development and Learning and Memory Disorders, Children’s Hospital of Chongqing Medical University, Chongqing, 400014 PR China; 2 Department of Anatomy, Basic Medical College, Chongqing Medical University, Chongqing, 400016 PR China; 3 Townsend Family Laboratories, Department of Psychiatry, The University of British Columbia, Vancouver, BC V6T 1Z3 Canada; 4 West China School of Basic Medical Sciences and Forensic Medicine, Sichuan University, Chengdu, 610041 Sichuan China; 5 Brain Research Centre, The University of British Columbia, Vancouver, BC V6T 2B5 Canada

**Keywords:** Diseases of the nervous system, Molecular neuroscience

## Abstract

Mitogen-activated protein kinase (MAPK) phosphatase 1 (MKP-1) is an essential negative regulator of MAPKs by dephosphorylating MAPKs at both tyrosine and threonine residues. Dysregulation of the MAPK signaling pathway has been associated with Alzheimer’s disease (AD). However, the role of MKP-1 in AD pathogenesis remains elusive. Here, we report that MKP-1 levels were decreased in the brain tissues of patients with AD and an AD mouse model. The reduction in MKP-1 gene expression appeared to be a result of transcriptional inhibition via transcription factor specificity protein 1 (Sp1) *cis*-acting binding elements in the MKP-1 gene promoter. Amyloid-β (Aβ)-induced Sp1 activation decreased MKP-1 expression. However, upregulation of MKP-1 inhibited the expression of both Aβ precursor protein (APP) and β-site APP-cleaving enzyme 1 by inactivating the extracellular signal-regulated kinase 1/2 (ERK)/MAPK signaling pathway. Furthermore, upregulation of MKP-1 reduced Aβ production and plaque formation and improved hippocampal long-term potentiation (LTP) and cognitive deficits in APP/PS1 transgenic mice. Our results demonstrate that MKP-1 impairment facilitates the pathogenesis of AD, whereas upregulation of MKP-1 plays a neuroprotective role to reduce Alzheimer-related phenotypes. Thus, this study suggests that MKP-1 is a novel molecule for AD treatment.

## Introduction

Alzheimer’s disease (AD) is an age-related neurodegenerative disease that leads to dementia and is characterized by extracellular senile plaques, intracellular neurofibrillary tangles and synaptic abnormalities. Amyloid-β (Aβ) peptides, the main component of senile plaques, are generated from Aβ precursor protein (APP) by sequential proteolytic cleavages by β- and γ-secretases.^1^ β-Site APP-cleaving enzyme 1 (BACE1) cleaves APP at the Asp^1^ or Glu^2^ site to generate a membrane-bound C-terminal fragment (CTF) of 99 amino acids (C99) or 89 amino acids (C89).^3–5^ C99 is further cleaved by γ-secretase to produce Aβ and the APP intracellular domain. Notably, in addition to the long-known secretases, recent studies have discovered that APP can be cleaved at a novel cleavage site termed η-site to produce CTF-η,^6,7^ which is partially mediated by membrane-bound matrix metalloproteinases such as MT5-MMP and is known as η-secretase activity. Then, CTF-η is cleaved by α- and β-secretases to release the long and short Aη peptide, including Aη-α and Aη-β, respectively.^6,8^ Previous studies have revealed that Aβ deregulates neurotransmitter release from the presynaptic site in both primary neurons and AD model mouse brains.^9–11^ Aβ directly interacts with cell membranes and membrane receptors to exert its neurotoxic effect and then initiates spine density decrease, synapse loss, and synaptic plasticity impairment,^2,12,13^ which may lead to neuronal perturbations and memory decline during AD.

Mitogen-activated protein kinases (MAPKs), which are serine/threonine protein kinases, play critical roles in cellular signal transduction. In mammals, MAPKs are comprised of extracellular signal-regulated kinase 1/2 (ERK), P38 kinases (P38), and c-Jun N-terminal kinases (JNK).^14^ In AD mouse models, the JNK signaling pathway is overactivated in the spine before cognitive decline, and its specific inhibitor D-JNKI1 is able to suppress synaptic shrinkage and postsynaptic protein loss.^15^ In the brains of AD patients, P38 is highly expressed.^16^ Aβ-induced P38 activation increases tau phosphorylation^17^ and promotes the amyloidogenic processing of APP.^18^ In the adult nervous system, ERK activation is required for synaptic plasticity and memory formation.^19,20^ However, overactivated ERK is associated with neurofibrillary tangle formation and early AD-related protein deposition, leading to hippocampal function impairment and memory deficits in both AD patients and mouse models.^21–24^ Collectively, these studies indicate that MAPKs could accelerate AD development. Prevention of MAPK overactivation can reduce Aβ deposition, tau hyperphosphorylation,^25^ neuronal apoptosis,^26^ and memory impairment.^27^ MAPKs could be potential targets for novel and effective therapeutics of AD.

MAPK phosphatase 1 (MKP-1) inhibits MAPK activity by dephosphorylating MAPK at the tyrosine and threonine residues.^28–30^ Although MKP-1 is widely expressed in rodent brain regions, including the hippocampus,^31^ cortex,^32^ ventral tegmental area,^33^ striatum and thalamus,^34^ its neurological function is poorly understood. Recent studies have shown that MKP-1 may serve as a pivotal regulator of synaptogenesis. Dysregulation of MKP-1 might disrupt neuronal development and cognitive function.^35^ A recent study has shown that MKP-1 exerts a neuroprotective role in Aβ-induced apoptosis, neuroinflammation, and oxidative stress by inactivating JNK.^36^ However, the exact role of MKP-1 in AD pathogenesis remains largely unclear.

In this study, we determined that MKP-1 levels were reduced in the brain tissues of patients with AD and a mouse model of AD. This reduction was associated with Aβ-induced Sp1 activation. Furthermore, we found that inhibition of the ERK/MAPK signaling pathway by MKP-1-reduced APP and BACE1 expression to generate Aβ, resulting in the inhibition of plaque formation, improvement of hippocampal long-term potentiation (LTP) and memory decline in APP/PS1 double transgenic mice. Our work demonstrates the neuroprotective effect of MKP-1 for potential AD treatment.

## Results

### Reduced MKP-1 expression in the brains of AD patients and a mouse model of AD

**Fig. 1:**
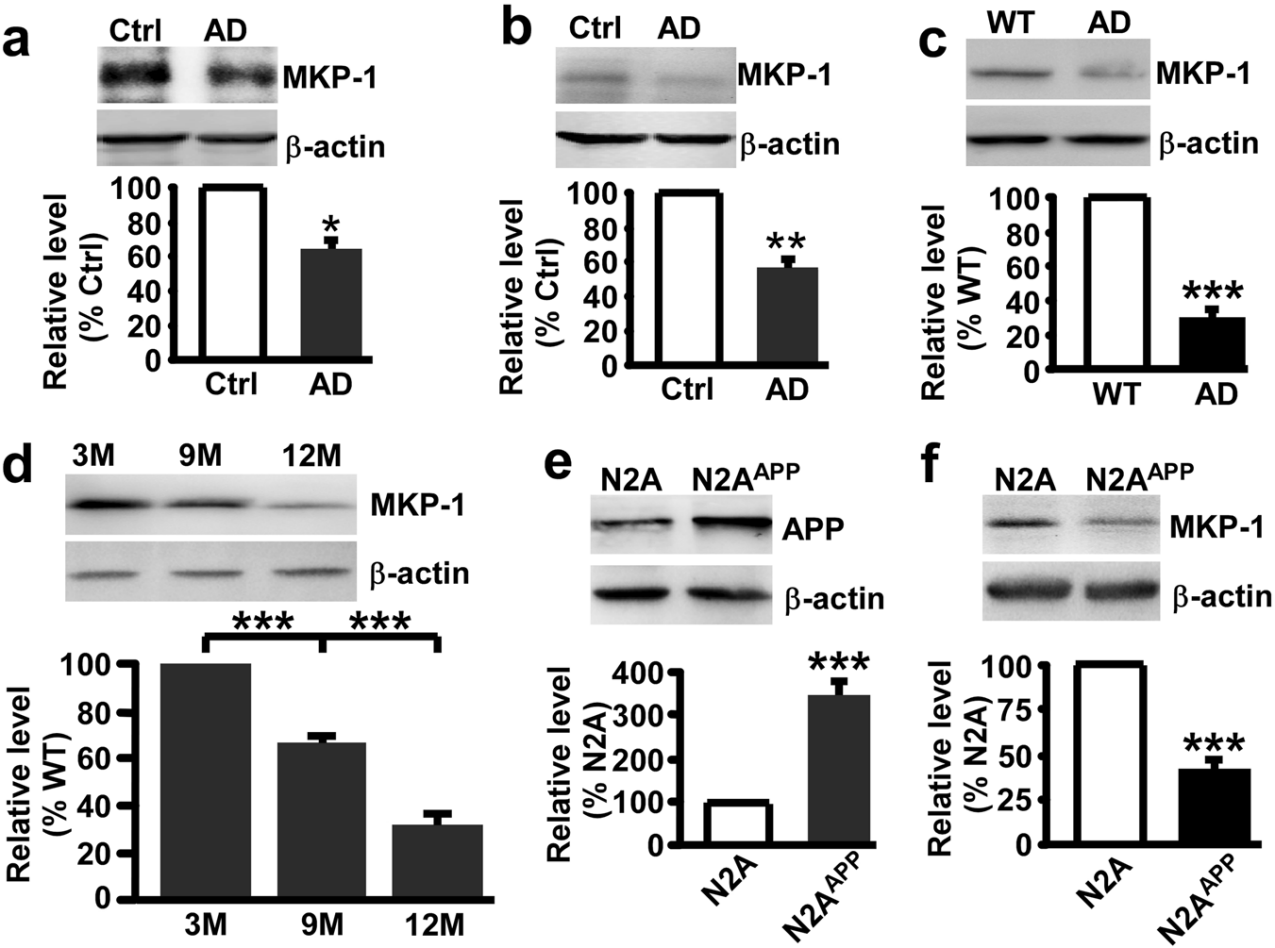
MKP-1 was decreased in AD. **a**, **b** The protein level of MKP-1 assessed by western blot in the hippocampus **a** and temporal cortex **b** of control (Ctrl) and AD patients. *p *<* 0.05 by unpaired Student’s *t* test. *n* = 4–6 in each group. **c** The protein level of MKP-1 in the hippocampus of wild-type (WT) and APP/PS1 transgenic AD model mice at the age of 9 months. ***p *<* 0.001 by unpaired Student’s *t* test. *n* = 6 in each group. **d** The protein level of MKP-1 in the hippocampus of AD mice at different ages. ***p *<* 0.001 by one-way ANOVA. *n* = 3 in each group. **e**, **f** The protein levels of APP **e** and MKP-1 **f** assessed by western blot in lysates of N2A and N2A^APP^ cells. ***p *<* 0.001 by unpaired Student’s *t* test. *n* = 3–6 in each group.

To examine whether there is an alteration of MKP-1 levels in AD, brain samples from patients with AD and control subjects were analyzed. The results showed that the expression of MKP-1 was markedly decreased in the hippocampus of AD patients (*n* = 6, 64.98 ± 10.77%, *p* *=* 0.023; Fig. 1a) relative to controls (*n* = 4). In addition to the hippocampus, other brain regions also play critical roles in AD development. Therefore, we next tested MKP-1 expression in the temporal cortex of human tissues and found that MKP-1 expression was also markedly decreased in AD patients (*n* = 6, 55.95 ± 10.94%, *p* *=* 0.009; Fig. 1b) relative to controls (*n* = 4). Next, we examined MKP-1 expression in the APP/PS1 double transgenic mouse model of AD. Consistent with previous reports,^31–34^ MKP-1 was widely distributed throughout the brain tissues of APP/PS1 transgenic mice (Supplementary Fig. S1). Similar to the findings in AD patients, MKP-1 expression was significantly reduced in the hippocampus of AD mice at 9 months old (28.46 ± 5.63%, *p* *<* 0.001; Fig. 1c) compared with their wild-type littermates. In addition, the reduction in MKP-1 in AD mice occurred in an age-dependent manner (66.79 ± 2.74% at 9 m and 32.31 ± 4.45% at 12 m relative to 3 m; Fig. 1d). We further examined whether mutant APP affects the expression of MKP-1 in N2A cells stably expressing human Swedish mutant APP695 (N2A^APP^). Swedish mutant APP was highly expressed in N2A^APP^ stable cells (349.17 ± 34.39%, *p* < 0.001; Fig. 1e) compared with N2A cells. Consistent with the downregulation of MKP-1 in both AD patients and AD mice, MKP-1 expression was also significantly decreased in N2A^APP^ cells (40.65 ± 5.98%, *p* < 0.001; Fig. 1f) compared with non-Swedish mutant APP-expressing cells. These data clearly showed that MKP-1 expression was decreased in AD, as observed in AD patients, a mouse model of AD and cells expressing the AD-associated mutant APP gene.

### Transcriptional downregulation of MKP-1 gene expression by Sp1

**Fig. 2:**
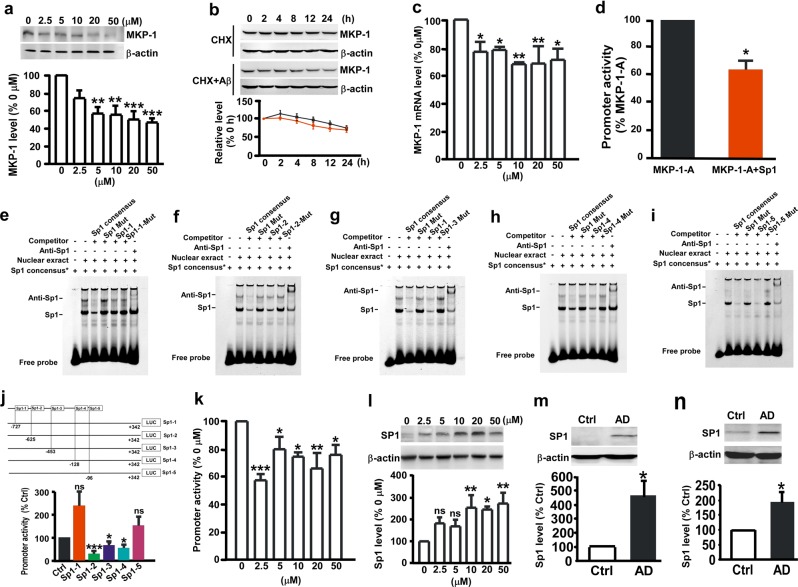
Sp1 inhibited MKP-1 expression in N2A^APP^ cells. **a** The protein level of MKP-1 assessed by western blot in N2A cells after treatment with different concentrations of Aβ. **p < 0.01 and ***p < 0.001 by one-way ANOVA. *n* = 5 in each group. **b** The effect of Aβ (10 µm) on the degradation of MKP-1 assessed by half-life measurements in N2A cells treated with 100 μg/ml cycloheximide (CHX). *p* = 0.352 by two-way ANOVA. *n* = 5–8 in each group. **c** The mRNA level of MKP-1 assessed by qPCR in N2A cells after treatment with different concentrations of Aβ. *p < 0.05 and **p < 0.01 by one-way ANOVA. *n* = 6 in each group. **d** The promoter activity of MKP-1 assessed by luciferase assay in N2A cells after treatment with different concentrations of Aβ. *p < 0.05, **p < 0.01 and ***p < 0.001 by one-way ANOVA. *n* = 5 in each group. **e–i** Functional Sp1-binding sites to the MKP-1 gene promoter. EMSA with MKP-1 Sp1 probe in nuclear extract of HEK 293 cells transfected with Sp1 expression plasmid. Lane 1 is the labeled human consensus Sp1 probe only. Lane 2 shows a shifted DNA–protein complex formed between the labeled Sp1 and nuclear extracts. Competition assays were performed by further adding different competitions of oligonucleotides that included consensus wild-type Sp1 (lane 3), mutant Sp1 (lane 4), putative Sp1-binding site 1 in MKP-1 and mutant Sp1-binding site 1 in MKP-1 (lanes 5 and 6 in a), putative Sp1-binding site 2 in MKP-1 and mutant Sp1-binding site 2 in MKP-1 (lanes 5 and 6 in **b**), putative Sp1-binding site 3 in MKP-1 and mutant Sp1-binding site 3 in MKP-1 (lanes 5 and 6 in **c**), putative Sp1-binding site 4 in MKP-1 and mutant Sp1-binding site 4 in MKP-1 (lanes 5 and 6 in **d**), putative Sp1-binding site 5 in MKP-1 and mutant Sp1-inding site 5 in MKP-1 (lanes 5 and 6 in **e**). Lane 7 shows the supershifted band with the anti-Sp1 antibody. **j**, **k** Effects of different Sp1-binding sites in MKP-1 on the promoter activity of MKP-1 as assessed by luciferase assay. *p < 0.05, **p < 0.01 and ***p < 0.001 by one-way ANOVA. *n* = 4 in each group. **l** Effect of different concentrations of Aβ on the expression of Sp1 as assessed by western blot in HEK 293 cells. *p < 0.05 and **p < 0.01 by one-way ANOVA. *n* = 4 in each group. **m**, **n** The protein level of Sp1 assessed by western blot in the hippocampus **m** and temporal cortex **n** of control (Ctrl) and AD patients. *p *<* 0.05 by unpaired Student’s *t* test. *n* = 4 to 6 in each group.

To determine whether the MKP-1 reduction in AD is attributed to Aβ, N2A cells were treated with different concentrations of Aβ. The results showed that Aβ treatment markedly decreased MKP-1 protein levels in a dose-dependent manner (74.59 ± 7.72% at 2.5 µm; 55.87 ± 6.10% at 5 µm; 54.95 ± 9.1% at 10 µm; 49.21 ± 8.04% at 20 µm; and 45.63 ± 5.10% at 50 µm; Fig. 2a). To determine whether the Aβ-induced reduction in MKP-1 is owing to impaired synthesis or enhanced degradation, cycloheximide (CHX) assay was performed. CHX was added to the cells to inhibit protein synthesis, and the MKP-1 protein level was analyzed. We found that 10 µm Aβ treatment had no effect on MKP-1 catabolism (Fig. 2b). However, Aβ treatment significantly decreased MKP-1 mRNA levels (Fig. 2c). These results indicate that Aβ inhibits MKP-1 gene expression at the transcriptional level but does not affect protein degradation.

To examine the transcriptional regulation of MKP-1 gene expression, human MKP-1 promoter plasmids were constructed that contained different lengths of the MKP-1 promoter upstream of the firefly luciferase reporter gene in the promoter-less vector pGL4.10-basic (Supplementary Fig. S2a). HEK 293 cells were transfected with these plasmids, and a luciferase assay was performed to examine the promoter activity.^37^ MKP-1-A, containing the longest MKP-1 promoter fragments from −763 bp to +342 bp of the transcription start site at +1, had robust promoter activity. Deletion of 534 bp at the 5’-flanking region from −763 to −229 resulted in significantly higher promoter activity, indicating that there are negative regulatory *cis*-acting element(s) in the −763 to −229 region. The deletion of 120 bp from +342 to +222 at the 3’-flanking region of the promoter had little effect on MKP-1 promoter activity. There was no promoter activity in the −47 to +342 or +222 region, but there was a high promoter activity in the region from −229 to −47, indicating that the region between −229 and −47 contains a minimal sequence for the MKP-1 promoter (Supplementary Fig. S2b).

Using Genomatix and TFSearch software to analyze potential regulatory *cis*-acting elements in the MKP-1 promoter, several potential Sp1-binding sites were identified in the MKP-1 promoter. To examine whether Sp1 regulates transcriptional activation of the MKP-1 gene promoter, the MKP-1 promoter plasmid MKP-1-A and Sp1 expression plasmid were cotransfected into HEK 293 cells. Compared with the control vector transfection, coexpression of Sp1 significantly decreased MKP-1 promoter activity (Fig. 2d). Electrophoretic mobility shift assay (EMSA) was performed, and the results showed that there were five Sp1-binding sites in the MKP-1 promoter (Fig. 2e–i). To examine whether all or some binding sites are physiologically functional in regulating transactivation of the MKP-1 promoter, the sites were abolished by mutations. Mutations in binding sites 2, 3, and 4 significantly decreased MKP-1 promoter activity to 28.49 ± 8.60%, 64.17 ± 12.45%, and 53.28 ± 13.22%, respectively, whereas mutations in binding sites 1 and 5 did not affect MKP-1 promoter activity (Fig. 2j). These results demonstrate that Sp1 negatively regulates the transcriptional activation of MKP-1 gene expression.

### Sp1 mediates Aβ-induced inhibition of MKP-1 expression

To investigate whether the Aβ-induced reduction in MKP-1 expression is caused by its inhibitory effect on the transcriptional activation of the MKP-1 gene, HEK 293 cells were transfected with the MKP-1 gene promoter plasmid MKP-1-A and then treated with Aβ. We found that Aβ treatment significantly reduced the promoter activity of MKP-1 (57.40 ± 4.41% at 2.5 µm; 79.89 ± 8.58% at 5 µm; 74.32 ± 3.20% at 10 µm; 75.97 ± 6.75% at 20 µm; and 75.63 ± 3.49% at 50 µm; Fig. 2k). As we showed that Sp1 transcriptionally inhibited MKP-1 gene expression, we next wanted to examine whether Sp1 mediates the inhibitory effect of Aβ on MKP-1 expression. The results showed that after treatment with different concentrations of Aβ the Sp1 protein level was increased in HEK 293 cells to 254.93 ± 58.08% at 10 µm, 246.35 ± 15.20% at 20 µm, and 275.11 ± 48.63% at 50 µm (Fig. 2l). Consistent with this result, Sp1 expression was markedly increased in the hippocampus (457.51 ± 112.89%, *p* *=* 0.025; Fig. 2m) and temporal cortex (190.51 ± 33.85%, *p* *=* 0.033; Fig. 2n) of AD patients (*n* = 6) relative to controls (*n* = 4). These data demonstrate that Aβ increases Sp1 expression and downregulates MKP-1 expression.

### MKP-1 affects APP processing by regulating APP and BACE1 expression

**Fig. 3:**
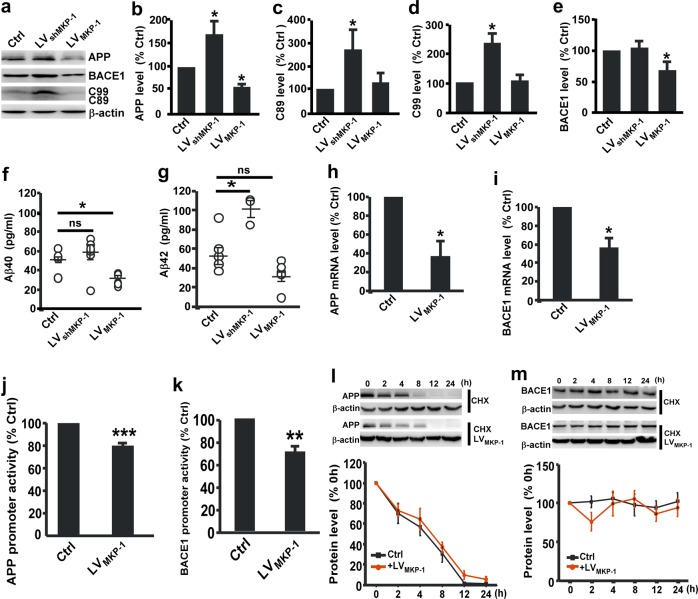
MKP-1 reduced APP processing in N2A^APP^ cells. **a**–**e** The protein levels of APP **b**, C89 **c**, C99 **d**, and BACE1 **e**, assessed by western blot in lysates of N2A^APP^ cells after overexpression of MKP-1 by LV_MKP-1_ or knockdown of MKP-1 by LV_shMKP-1_. *p < 0.05 by one-way ANOVA. *n* = 5–6 in each group. **f**, **g** The generation of Aβ_40_ **f** and Aβ_42_ **g** as measured by ELISA in the conditioned media of N2A^APP^ cells after overexpression of MKP-1 by LV_MKP-1_ or knockdown of MKP-1 by LV_shMKP-1_. *p < 0.05 by one-way ANOVA. *n* = 5–6 in each group. **h**, **i** The mRNA levels of APP **h** and BACE1 **i** as assessed by qPCR in N2A^APP^ cells after LV_MKP-1_ transfection. *p = 0.017 for APP and *p = 0.038 for BACE1 by unpaired Student’s *t* test. *n* = 3–4 in each group. **j**, **k** The promoter activity of APP **j** and BACE1 **k** assessed by luciferase assay in cells transfected with LV_MKP-1_. ***p < 0.001 and **p = 0.004 by unpaired Student’s *t* test. *n* = 3–4 in each group. **l**, **m** The degradation of APP **l** and BACE1 **m** assessed by half-life measurements in LV_MKP-1_-transfected N2A^APP^ cells treated with 100 μg/ml cycloheximide (CHX). *p* = 0.438 for APP and *p* *=* 0.483 for BACE1 by two-way ANOVA. *n* = 4–5 in each group.

Our study has shown that MKP-1 expression is decreased in the brain tissues of AD patients and mice. To further explore whether MKP-1 affects APP processing and Aβ generation, N2A^APP^ cells were infected with lentivirus carrying the MKP-1 gene (LV_MKP-1_). Overexpression of MKP-1 significantly decreased the levels of APP to 57.69 ± 6.47% (*p* *=* 0.044, Fig. 3a, b) and the β-secretase BACE1 to 68.98 ± 12.34% (*p* *=* 0.030, Fig. 3a, e). Aβ_40_ and Aβ_42_ were also significantly reduced to 27.81 ± 2.42 pg/ml (Fig. 3f) and 33.18 ± 5.32 pg/ml (Fig. 3g), respectively. In contrast, downregulation of MKP-1 expression by MKP-1 shRNA (LV_shMKP-1_) markedly increased the expression of APP to 171.78 ± 28.19% (*p* *=* 0.021) and its CTFs C89 to 270.83 ± 83.08% (*p* *=* 0.023; Fig. 3a, c) and C99 to 226.48 ± 30.18% (*p* *=* 0.036; Fig. 3a, d). Aβ_40_ and Aβ_42_ increased to 54.69 ± 7.80 pg/ml (Fig. 3f) and to 109.7 ± 9.54 pg/ml (Fig. 3g), respectively.

To examine whether MKP-1 regulates APP and BACE1 gene expression, we first measured the APP and BACE1 mRNA levels in N2A^APP^ cells. We found that overexpression of MKP-1 by the MKP-1 lentivirus significantly decreased the levels of APP mRNA to 56.68 ± 10.20% (*p* *=* 0.017; Fig. 3h) and BACE1 mRNA to 37.22 ± 15.82% (*p* *=* 0.038; Fig. 3i). The promoter activities of APP and BACE1 were further assayed by cotransfecting N2A^APP^ cells with the human APP promoter- or human BACE1 promoter-containing plasmid and MKP-1 lentivirus. Luciferase assays showed that MKP-1 overexpression significantly decreased the APP promoter activity of pAPP-Luc (79.33 ± 18.45% relative to control, *p* *<* 0.001; Fig. 3j) and BACE1 promoter activity of pB1-A-Luc (70.95 ± 3.91% relative to control, *p* *=* 0.004; Fig. 3k). To detect whether MKP-1 affects the degradation of the APP and BACE1 proteins, a CHX assay was performed. We observed that MKP-1 did not affect the degradation of APP (Fig. 3l) or BACE1 (Fig. 3m). These results suggest that MKP-1 downregulates APP and BACE1 expression and APP processing to generate Aβ.

### MKP-1 inhibits the amyloidogenic process through the ERK/MAPK signaling pathway

**Fig. 4:**
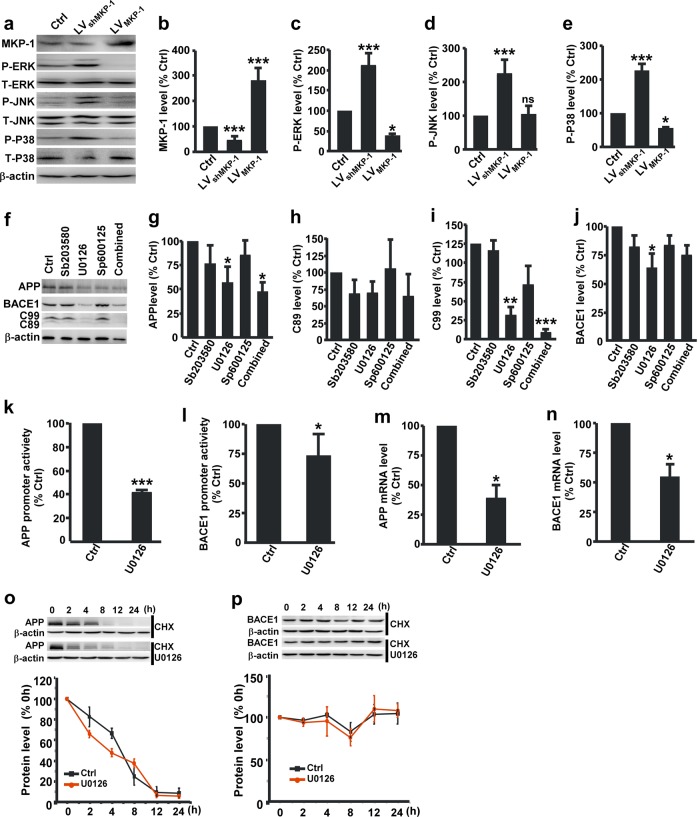
MKP-1 inhibited APP processing through inactivation of the ERK/MAPK signaling pathway. **a**–**e** Immunoblot of the expression of MKP-1 **b**, P-ERK **c**, P-JNK **d** and P-P38 **e** in lysates of N2A^APP^ cells after overexpression of MKP-1 by LV_MKP-1_ or knockdown of MKP-1 by LV_shMKP-1_. *p < 0.05 and ***p < 0.001 by one-way ANOVA. *n* = 4–5 in each group. **f**–**j** Immunoblot of the expression of APP **g**, C89 **h**, C99 **i** and BACE1 **j** in lysates of N2A^APP^ cells treated with the ERK inhibitor U0126, the JNK inhibitor SP600125, the P38 inhibitor SB203580 or the three inhibitors together (Combined). *p < 0.05, **p < 0.01 and ***p < 0.001 by one-way ANOVA. *n* = 5–7 in each group. **k**, **l** The mRNA levels of human APP **k** and BACE1 **l** assessed by qPCR in N2A^APP^ cells and N2A^APP^ cells treated with U0126. ***p < 0.001 for APP and *p = 0.032 for BACE1 by unpaired Student’s *t* test. *n* = 3–4 in each group. **m**, **n** The promoter activity of APP **m** and BACE1 **n** assessed by luciferase assay in N2A^APP^ cells and N2A^APP^ cells treated with U0126. *p = 0.036 for APP and *p = 0.040 for BACE1 by unpaired Student’s *t* test. *n* = 3–4 in each group. **o**, **p** The degradation of APP **o** and BACE1 **p** as assessed by half-life measurements in the N2A^APP^ cells after U0126 treatment. *p* = 0.264 for APP and *p* = 0.847 for BACE1 by two-way ANOVA. *n* = 4–5 in each group.

Next, we detected the influence of MKP-1 on ERK, P38 and JNK. The results showed that P-ERK, P-P38 and P-JNK were significantly increased in N2A^APP^ cells compared with N2A control cells (Supplementary Fig. S3). To examine the role of MKP-1 in MAPK activation, LV_MKP-1_ or LV_shMKP-1_ were transfected into N2A^APP^ cells to overexpress or knockdown MKP-1, respectively. MKP-1 knockdown led to a significant increase in P-ERK (208.82 ± 34.00% relative to control, *p* *<* 0.001), P-JNK (223.46 ± 43.63%, *p* *<* 0.001) and P-P38 (226.65 ± 20.28% relative to control, *p* *<* 0.001) (Fig. 4a–e). In contrast, overexpression of MKP-1 significantly decreased P-ERK (38.71 ± 3.25% relative to control, *p* *=* 0.035) and P-P38 (56.33 ± 3.90% relative to control, *p* *=* 0.024) but not P-JNK (105.83 ± 25.13% relative to control, *p* *=* 0.998) (Fig. 4a–e). Furthermore, treatment with an ERK inhibitor (U0126) but not JNK inhibitor (SP600125) or P38 inhibitor (SB203580) significantly decreased the expression of APP, C89, C99 and BACE1 (Fig. 4f–j). In addition, the combined application of U0126, SP600125 and SB203580 had the same effect on APP processing as U0126 treatment alone (Fig. 4f–j), suggesting that inhibition of the ERK/MAPK signaling pathway is sufficient to inhibit the amyloidogenic processing of APP. Furthermore, U0126 markedly inhibited the promoter activities of APP to 41.28 ± 2.52% (*p* *<* 0.001; Fig. 4k) and BACE1 to 67.52 ± 10.83% (*p* *=* 0.040; Fig. 4l) and reduced the APP mRNA levels down to 38.06 ± 12.05% relative to control (*p* = 0.036; Fig. 4m) and BACE1 mRNA level down to 54.35 ± 10.45% (*p* *=* 0.032; Fig. 4n). U0126 treatment did not affect APP or BACE1 protein degradation (Fig. 4o, p). Our results clearly demonstrate that MKP-1 inhibits APP and BACE1 gene expression and the amyloidogenic processing of APP through the ERK/MAPK signaling pathway.

### MKP-1 reduces Aβ generation and plaque formation in APP/PS1 mice

**Fig. 5:**
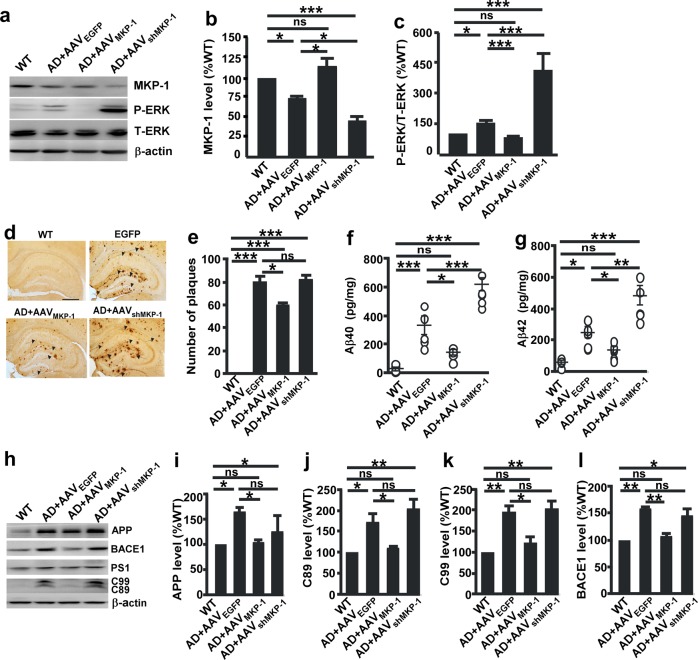
MKP-1 reduced Aβ generation and senile plaque numbers in APP/PS1 mice. **a**–**c** Immunoblot of the expression of MKP-1 **b** and P-ERK **c** in the hippocampal homogenates of wild-type (WT) and APP/PS1 transgenic AD mice after overexpression of MKP-1 by AAV_MKP-1_ or knockdown of MKP-1 by AAV_shMKP-1_. *p < 0.05, **p < 0.01 and ***p < 0.001 by one-way ANOVA. *n* = 4–5 in each group. **d**, **e** The number of senile plaques detected by immunohistochemistry in the hippocampus of AD model mice after overexpression of MKP-1 by AAV_MKP-1_ or knockdown of MKP-1 by AAV_shMKP-1_. *p < 0.05 and ***p < 0.001 by one-way ANOVA. n = 29 to 54 in each group. **f**, **g** Generation of Aβ_40_ **f** and Aβ_42_ **g** as measured by ELISA in the hippocampal homogenates of WT and AD model mice after overexpression of MKP-1 by AAV_MKP-1_ or knockdown of MKP-1 by AAV_shMKP-1_. *p < 0.05, **p < 0.01, and ***p < 0.001 by one-way ANOVA. *n* = 5 in each group. **h**–**l** Immunoblot of the expression of APP **i**, C89 **j**, C99 **k** and BACE1 **l** in the hippocampal homogenates of WT and AD mice after overexpression of MKP-1 by AAV_MKP-1_ or knockdown of MKP-1 by AAV_shMKP-1_. *p < 0.05 and **p *<* 0.01 by one-way ANOVA. *n* = 5 in each group.

To determine the role of the MKP-1-mediated ERK/MAPK signaling pathway in AD pathogenesis and its therapeutic potential, we generated an adeno-associated viruses carrying MKP-1 cDNA (AAV_MKP-1_) and MKP-1 shRNA (AAV_shMKP-1_). The viruses were microinjected into the lateral ventricle of APP/PS1 mice to overexpress or knockdown MKP-1 in the AD mouse model (Fig. 5a, b). Consistent with the observations in N2A^APP^ cells, the P-ERK level was significantly increased in AD mice (155.93 ± 24.54%, *p* *=* 0.034; Fig. 5a, c) compared with WT mice. Overexpression of MKP-1 by AAV_MKP-1_ inhibited P-ERK in AD mice (*p* < 0.001), whereas downregulation of MKP-1 expression by AAV_shMKP-1_ increased P-ERK (*p* *<* 0.001) (Fig. 5a, c).

Neuritic plaques are a pathological hallmark of AD. Our results have shown the effect of MKP-1 on APP processing and Aβ generation in vitro and its underlying mechanism. To confirm the effect in vivo, APP processing and Aβ generation were assayed in the AD transgenic mouse model infected with AAV_MKP-1_ or AAV_shMKP-1_. Overexpression of MKP-1 by AAV_MKP-1_ in the brains of AD mice decreased the expression of APP (AAV_EGFP_: 221.22 ± 57.47% vs. AAV_MKP-1_: 117.09 ± 23.97%, *p* = 0.037), C89 (AAV_EGFP_: 169.42 ± 29.51% vs. AAV_MKP-1_: 106.44 ± 15.11%, *p* *=* 0.039), C99 (AAV_EGFP_: 195.15 ± 35.38% vs, AAV_MKP-1_: 121.52 ± 33.51%, *p* = 0.016), and BACE1 (AAV_EGFP_: 156.70 ± 13.56% vs. AAV_MKP-1_: 105.18 ± 17.36%, *p* = 0.005) (Fig. 5h–l). However, the expression of APP, C89, C99 and BACE1 was not affected by MKP-1 knockdown with AAV_shMKP-1_ (Fig. 5h–l).

Overexpression of MKP-1 by AAV_MKP-1_ led to a marked reduction in the levels of Aβ_40_ (WT: 21.56 ± 11.44 pg/mg; AD + AAV_EGFP_: 288.67 ± 56.03 pg/mg; and AD + AAV_MKP-1_: 125.30 ± 39.61 pg/mg) (*p* *=* 0.025; Fig. 5f) and Aβ_42_ (WT: 52.70 ± 10.40 pg/mg; AD + AAV_EGFP_: 221.15 ± 34.02 pg/mg; and AD + AAV_MKP-1_: 121.03 ± 22.95 pg/mg) (*p* *=* 0.049; Fig. 5g) in the mouse brains. In contrast, knockdown of MKP-1 by AAV_shMKP-1_ increased the levels of Aβ_40_ (AD + AAV_shMKP-1_: 539.84 ± 88.00 pg/mg) (*p* *<* 0.001; Fig. 5f) and Aβ_42_ (AD + AAV_shMKP-1_: 425.10 ± 53.85 pg/mg) (*p* *=* 0.003; Fig. 5g). To determine whether MKP-1 affects AD-related neuropathologies, the formation of neuritic plaques was examined in the APP/PS1 mice. Overexpression of MKP-1 by AAV_MKP-1_ significantly decreased the number of neuritic plaques (*p* *=* 0.028; Fig. 5d, e). However, downregulation of MKP-1 expression by AAV_shMKP-1_ had no effect on plaque formation. Collectively, these data demonstrate that overexpression of MKP-1 inhibits Aβ generation and neuritic plaque formation in AD transgenic mice.

### MKP-1 alleviates synaptic and cognitive impairments in APP/PS1 mice

**Fig. 6:**
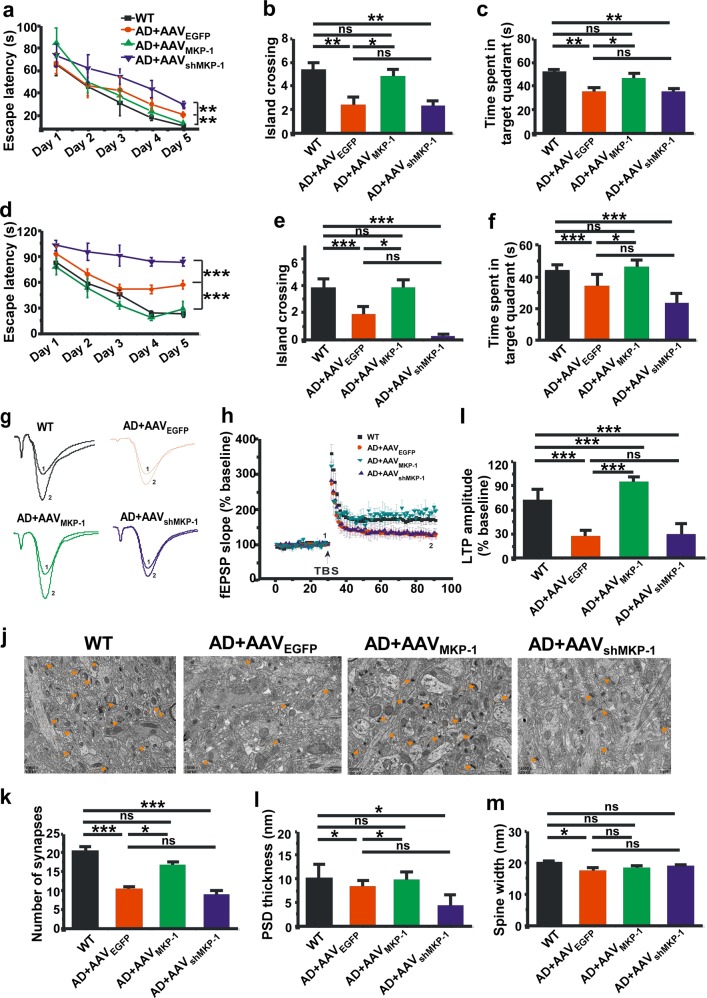
MKP-1 alleviated synaptic and memory deficits in APP/PS1 mice. **a**–**c** Spatial learning and memory assessed by the Morris water maze test in wild-type (WT) and APP/PS1 transgenic AD mice at the age of 9 months after overexpression of MKP-1 by AAV_MKP-1_ or knockdown of MKP-1 by AAV_shMKP-1_. The escape latency to the hidden platform **a** during the spatial learning period. **p < 0.01 by two-way ANOVA. The number of entries into the platform zone **b** and the time spent in the hidden platform-located quadrant **c** during the probe test in absence of the hidden platform in mice. *p < 0.05 and **p < 0.01 by one-way ANOVA. *n* = 7–12 in each group. **d**–**f** Spatial learning and memory as assessed by the Morris water maze test in WT and AD mice at the age of 12 months after overexpression of MKP-1 by AAV_MKP-1_ or knockdown of MKP-1 by AAV_shMKP-1_. The escape latency to the hidden platform **d** during the spatial learning period. ***p < 0.001 by two-way ANOVA. The number of entries into the platform zone **e** and the time spent in the hidden platform-located quadrant **f** during the probe test in absence of the hidden platform in mice. *p < 0.05 and ***p < 0.001 by one-way ANOVA. *n* = 10 in each group. **g**–**i** Hippocampal CA1 LTP recorded from brain slices of WT and AD mode mice at the age of 12 months after overexpression of MKP-1 by AAV_MKP-1_ or knockdown of MKP-1 by AAV_shMKP-1_. **p < 0.01 by one-way ANOVA. *n* = 4 to 10 slices from three mice in each group. **j**–**m** Transmission electron microscopic analysis was performed to examine the changes in synapse numbers **k**, PSD thickness **l** and spine width **m** in the hippocampus of WT and AD mice at the age of 12 months after overexpression of MKP-1 by AAV_MKP-1_ or knockdown of MKP-1 by AAV_shMKP-1_. *p < 0.05 and ***p < 0.001 by one-way ANOVA. *n* = 4 in each group.

Our study has indicated that overexpression of MKP-1 can ameliorate neuropathology in AD mice. We next wanted to detect whether MKP-1 could improve cognitive impairments. APP/PS1 mice were treated with AAV_MKP-1_ or AAV_shMKP-1_ at 3 and 6 months of age and subjected to the Morris water maze test at 9 months of age. Compared with WT mice, the transgenic mice treated with control AAV displayed significantly impaired spatial learning with longer escape latency for finding the hidden platform (*p* = 0.007; Fig. 6a). However, upregulation of MKP-1 by AAV_MKP-1_ markedly shortened the escape latency (*p* = 0.016; Fig. 6a), whereas downregulation of MKP-1 by AAV_shMKP-1_ increased the escape latency in the AD mice (*p* = 0.006; Fig. 6a). The probe test showed that upregulation of MKP-1 enhanced spatial memory retrieval, as the AAV_MKP-1_-treated mice had an increased number of entries into the platform zone (*p* *=* 0.017; Fig. 6b) and spent much more time in the target quadrant (*p* = 0.032; Fig. 6c). We also treated another group of mice at a later stage by microinjecting the AD mice with AAV at 6 and 9 months of age, and performed the behavioral test at 12 months. Similar results were observed. AAV_MKP-1_ decreased escape latency during hidden platform training (*p* < 0.001; Fig. 6d). The probe test revealed that mice treated with AAV_MKP-1_ had more entries into the platform zone (*p* *=* 0.037; Fig. 6e) and spent much more time in the target quadrant (*p* *=* 0.034; Fig. 6f). These data clearly demonstrate that upregulation of MKP-1 expression ameliorated the cognitive impairments in the AD transgenic mouse model.

Hippocampal LTP is considered to be the cellular mechanism underlying learning and memory. Next, we further investigated the influence of MKP-1 on hippocampal LTP in the CA1 area of AD mice at 12 months of age. We found that LTP was clearly impaired in APP/PS1 mice (127.79 ± 1.23% vs. 172.06 ± 2.24% in control to baseline, *p* < 0.001; Fig. 6g–i). Upregulation of MKP-1 by AAV_MKP-1_ reversed the impairment of LTP (194.68 ± 9.32% to baseline, *p* < 0.001), whereas AAV_shMKP-1_ treatment did not affect LTP in AD mice (Fig. 6g–i). In addition, we measured the effect of MKP-1 on synaptic structure changes in the AD mouse model. The total number of synapses was dramatically decreased in AD mice (10.94 ± 0.22 vs. 21.05 ± 1.57 in the control, *p* < 0.001; Fig. 6j, k). However, upregulation of MKP-1 expression by AAV_MKP-1_ significantly increased the total number of synapses to 18.85 ± 0.25, whereas knockdown of MKP-1 expression by AAV_shMKP-1_ further reduced the total number of synapses to 9.00 ± 2.36 in AD mice (*p* *<* 0.001; Fig. 6j, k). Furthermore, similar results were observed with the thickness of postsynaptic density (PSD). The thickness of PSD was 9.78 ± 0.63 nm in AD mice compared with 19.94 ± 1.34 nm in nontransgenic mice (*p* *=* 0.045). Upregulation of MKP-1 by AAV_MKP-1_ restored the thickness to 21.96 ± 4.01 nm (*p* = 0.018), whereas knockdown of MKP-1 by AAV_shMKP-1_ had no effect on thickness at 10.05 ± 0.95 nm in the hippocampal CA1 region of AD mice (Fig. 6j, l). Notably, no obvious difference was observed in the width of the synaptic cleft in mice treated with AAV_MKP-1_ or AAV_shMKP-1_ (Fig. 6j, m). Together, these findings suggest that upregulation of MKP-1 rescues synaptic deficits and ameliorates cognitive impairments in the AD model in vivo.

## Discussion

MKP-1 has essential roles in regulating neuronal growth and synaptogenesis by inhibiting MAPK signaling in the central nervous system. Here, we reported that MKP-1 expression was significantly reduced in the brains of patients with AD and in a mouse model of AD. We also found that MKP-1 transcriptional activation was regulated by Sp1. Aβ treatment decreased MKP-1 expression by upregulating Sp1. We demonstrated that MKP-1 regulated ERK/MAPK-mediated APP and BACE1 transcriptions, thus affecting APP processing and Aβ production. These findings reveal that inhibiting the MKP-1 signaling pathway could facilitate AD pathogenesis. Our results further suggested that MKP-1 upregulation reduced Aβ production and plaque formation, rescued synaptic abnormalities, and improved cognitive decline in AD mice (Supplementary Fig. 4). Our study clearly demonstrates the effect of MKP-1 on AD pathogenesis and its therapeutic potential for AD treatment.

MAPKs are involved in Aβ deposition,^21,38–40^ tau protein phosphorylation,^21^ and inflammatory responses.^41^ MKP-1, an inhibitor of MAPKs,^28–30^ may play an essential role in AD. The present study found that MKP-1 expression was obviously decreased in patients with AD and AD mice. We found that a reduction in MKP-1 resulted in the overactivation of the ERK/MAPK signaling pathway and thereby increased Aβ generation. Aβ is produced from APP via sequential cleavages by β- and γ-secretases.^1^ Dysregulation of BACE1 is involved in AD pathogenesis, and inhibition of BACE1 reduces Alzheimer’s phenotypes.^4,37,42^ Our data showed that upregulation of MKP-1 inhibited APP and BACE1 expression, leading to a reduction in the amyloidogenic processing of APP to generate Aβ. In contrast, downregulation of MKP-1 increased Aβ generation in AD mice. However, it is interesting that downregulation of MKP-1 did not affect APP or BACE1 expression or the number of neuritic plaques. One possibility is that reduced MKP-1 in AD models is sufficient to increase APP and BACE1 expression so that genetic knockdown of MKP-1 cannot induce further increases in APP and BACE1.

Growing evidence has demonstrated that ERK activation is required for synaptic plasticity and memory.^19,20^ Inhibition of ERK activation by the inhibitor SL327 causes significant long-term memory impairments.^43^ However, nonspecific ERK phosphorylation or overactivation may account, at least in part, for memory impairment owing to altered signaling in diseases. Previous studies have suggested that ERK is overactivated in AD,^21–23^ and decreasing ERK activation in the prefrontal cortex can reverse early memory decline in AD mice.^24^ Consistent with these findings, we here reported that AD mice displayed dramatically higher levels of activated ERK at 12 months of age. All members of the MAPK family have been implicated in AD.^21,38–40^ In this study, we demonstrated that MKP-1 reduction results in the overactivation of ERK, JNK and P38. However, our data showed that only the ERK/MAPK pathway has key roles in Aβ generation by inhibiting the transcription of the APP and BACE1 genes. One possibility is that JNK and P38 have little effect on BACE1-mediated APP processing, but they are able to participate in AD development through other molecular mechanisms. For instance, the JNK/MAPK signaling pathway can be activated in the synapse before the onset of cognitive impairment.^15^ Inhibition of JNK activity by CEP-1347 blocked the Aβ-induced neurotoxicity and downstream c-Jun and caspase-2 and −3 activation,^44^ consequently preventing synaptic shrinkage and postsynaptic protein loss.^45^ Aβ-induced oxidative stress can lead to P38/MAPK activation and tau hyperphosphorylation.^17^ In addition, Aβ stimulates rapid P38/MAPK activation, leading to inflammatory gene expression and proinflammatory cytokine release in microglia.^46,47^ Thus, inhibition of P38 overactivation with MW01-2-069A-SRM significantly attenuated the Aβ-induced increase in proinflammatory cytokines and reduced the Aβ-mediated synaptic and behavioral deficits in mice.^48^

In summary, we determined that upregulation of MKP-1 significantly inhibits the amyloidogenic processing of APP by regulating the ERK/MAPK signaling pathway, reduces the senile plaque number and ameliorates cognitive function in a mouse model of AD model. These findings provide novel insights into the role of MKP-1 in the pathogenesis of AD and its potential as a new target for AD therapy.

## Materials and methods

### Animals

APP/PS1 mice were obtained from Beijing HFK Bioscience Co. and reared in a temperature- and humidity-controlled specific-pathogen-free room (lights on from 7:00 a.m. to 7:00 p.m.) at Children’s Hospital of Chongqing Medical University. The genotype was confirmed by PCR using tail tissue DNA. All animal experiments were conducted in accordance with the Chongqing Science and Technology Commission guidelines and approved by the Animal Care Committee of Chongqing Medical University.

### Patient samples

Ten postmortem human brain samples, six of which were clinically diagnosed with AD, were obtained from the Chinese Human Brain Bank of Zhejiang University (Supplementary Table 1). These samples were pathologically confirmed by the Chinese Human Brain Bank. Brain tissues were homogenized in ice-cold TRIzol (Takara, Otsu, Shiga, Japan). Chloroform was added to separate the phases, and then an equal volume of isopropyl alcohol was mixed with the aqueous phase. After the pellet was washed with 70% ethanol, RNA was detected by a spectrophotometer NanoDrop 2000 (Nanodrop Technologies, Wilmington, DE, USA). Moreover, samples were homogenized in homogeneous buffer in a mortar and pestle and centrifuged (12,000 *g*, 4 °C for 15 min) to collect the supernatants for western blotting assay. The human study was evaluated and approved by the Ethics Committee of Zhejiang University (number of the research project ethical approval document: 2018-009).

### Antibodies

APP and its CTFs were detected by a polyclonal antibody C20 (1:1000) that was obtained from the laboratory of professor Weihong Song.^49^ Anti-MKP-1 (1:200, #sc-2857) was obtained from Santa Cruz Biotechnology. Anti-JNK (1:1000, #9252), anti-P-JNK (1:1000, #4668), anti-P38 (1:1000, #8690), anti-P-P38 (1:1000, #4511), anti-ERK (1:1000, #4695), anti-P-ERK (1:1000, #4370) and anti-BACE1 (1:1000, #5606) were purchased from CST. Anti-β-actin (1:3000, #A5411) antibody was purchased from Sigma.

### Plasmids

Genomic DNA extracted from HEK 293 cells was used to amplify the promoter region of human MKP-1 by PCR. From −763 bp to +342 bp of the transcription start site at +1, six fragment promoter regions of MKP-1 were amplified by PCR, and the luciferase reporter gene was inserted into the pGL4.10 expression vector (MKP-1-A, -B, -C, -D, -E, and -F; Supplementary Fig. S2). To construct different sequences of the MKP-1 promoter, the following primers were used: −763 fKpnI: 5′-CCGGGTACCAAAAGTCTGGGAAACAGGAAAG, −229 fKpnI: 5′-CCGGGTACCGCTCCGAGGCTGATGACGT, −47 fKpnI: 5′-CCGGGTACCGCTGCGAAGGACATTTGG, +222 rHindIII: 5′-TACAAGCTTCAGGGTGCCCACTTCCAT, +342 rHindIII: 5′-TACAAGCTTGAAGCGCACGTTGACAGAG, and −47 rHindIII: 5′-TACAAGCTTGAGCCTGGCCCGGGGAGCGCGTTTA.

The following series of substitution mutations of the Sp1-binding sites in MKP-1 were constructed: for Sp1-binding site 1 in MKP-1 (Sp1-1), TCTCCGCCCCAACTCG was mutated to AAAAAAAAAAAAAAAA; for Sp1-binding site 2 in MKP-1 (Sp1-2), CCCCCACCCCA was mutated to AAAAAAAAAAA; for Sp1-binding site 3 in MKP-1 (Sp1-3), AGCCCTCCTCCTCCCCG was mutated to AAAAAAAAAAAAAAAAA; for Sp1-binding site 4 in MKP-1 (Sp1-4), CCCCCCCTCCCCC was mutated to AAAAAAAAAAAAA; and for Sp1-binding site 5 in MKP-1 (Sp1-5), GGCCCGCCCCGTCCCCC was mutated to AAAAAAAAAAAAAAAAA (Supplementary Table 2).

### Cell culture and transfection

N2A cells were cultured in 47% Dulbecco’s modified Eagle’s medium (DMEM) (Gibco, New York, USA) and 47% Opti-MEM (Gibco, New York, USA), supplemented with 1% streptomycin and 5% fetal bovine serum (FBS). N2A^APP^ cells stably transfected with the human Swedish mutant APP695 were obtained from Professor Chunjiu Zhong (Fudan University, Shanghai, China) and cultured in complete DMEM containing G418 at a concentration of 100 µg/ml. Cells were maintained at 37 °C in a 5% CO_2_ atmosphere. The cells were seeded in six-well plates until grown to 30–40% confluence and then transfected with LV_MKP-1_ or LV_shMKP-1_ to produce a final MOI of 20. Approximately 8–12 h later, complete medium was added, and cells were harvested for western blotting 72 h after that. To overexpress or knockdown MKP-1 in vitro, lentivirus overexpressing MKP-1 (LV_MKP-1_) or MKP-1 carrying small hairpin RNA (LV_shMKP-1_) was constructed by OBiO Technology (Shanghai, China). siRNA against mouse MKP-1 was obtained from Santa Cruz Biotechnology, and the MKP-1 overexpression plasmid was synthetized by OBiO. The neuron-specific promoter P2A was used to drive MKP-1 expression, and the H1 promoter was used for shRNA-MKP-1.

### Adeno-associated virus and microinjection

To overexpress or knockdown MKP-1 in vivo, adeno-associated virus expressing MKP-1 (AAV_MKP-1_) or MKP-1 small hairpin RNA (AAV_shMKP-1_) was constructed by OBiO Technology. Titers were 3 × 10^12^ TU/ml. After anesthetization with sodium pentobarbital, mice were mounted on a stereotaxic instrument, and 1 μl of AAV_MKP-1_ or AAV_shMKP-1_ was microinjected into the lateral ventricle per hemisphere by a drilled hole (0.4 mm posterior, ±1 mm lateral and 3 mm ventral relative to bregma). All mice received two AAV microinjections before the behavioral test. Some mice received AAV microinjections at the age of 6 and 9 months, and the behavioral tests were performed at the age of 12 months. Other mice received AAV microinjections at the age of 3 and 6 months, and the behavioral tests were performed at the age of 9 months.

### Immunohistochemistry staining

After animals were killed with an overdose of urethane, one half of the mouse brain was immediately frozen for protein or RNA extraction. The other half of the brain was postfixed in freshly prepared 4% paraformaldehyde in phosphate-buffered saline (PBS, 0.1 m, pH 7.4) for 24 h, then dehydrated with 30% sucrose until the tissue sank to the bottom and serially sectioned into 30 μm-thick coronal sections. To eliminate residual peroxidase activity, the slices were incubated with 3% H_2_O_2_ for half an hour. Then, the slices were blocked with 10% bovine serum albumin and incubated with mouse monoclonal 4G8 antibody overnight at 4 °C. Plaques were detected by the ABC and DAB methods and counted by microscopy at × 40 magnification as described previously.^50^

### Quantitative real-time PCR

Total RNA was extracted from brain tissue or cells using TRIzol reagent (Takara, Otsu, Shiga, Japan), and the concentration and purity were detected with a spectrophotometer NanoDrop 2000 (Nanodrop Technologies, Wilmington, DE, USA). One microgram of RNA was used to synthesize the first-strand complementary DNA (cDNA) with the Prime Script RT reagent Kit (Takara, Otsu, Shiga, Japan). The cDNA of MKP-1, APP and BACE1 were analyzed by quantitative real-time PCR by using SYBR Premix Ex Taq II (Takara, Otsu, Shiga, Japan) with the CFX Manager software detection system (Bio-Rad). Primer sequences were as follows: MKP-1 (forward: 5′-GTACATAAGTCCATCTGAC, reverse: 5′-GGTTCTTCTAGGAGTAGACA); APP (forward: 5′-ATGCCGTTGACAAGTATCTCG, reverse: 5′-TCTGCCTCTTCCCATTCTCTC); BACE1 (forward: 5′-TACCAACCAGTCCTTCCGC, reverse: 5′-CTCCCATAACAGTGCCCGT); and GAPDH (forward: 5′-AACTGCTTAGCACCCCTGGC, reverse: 5′-ATGACCTTGCCCACAGCCTT). The relative expression levels of MKP-1, APP and BACE1 cDNAs were normalized to GAPDH levels.

### Western blot assay

The hippocampus and temporal cortex were lysed in homogeneous buffer in a mortar and pestle. The homogenates were centrifuged (4 °C, 10,000 rpm, 15 min) to collect the supernatants. Protein samples (30 μg) were boiled in 4 × loading buffer at 95 °C for 10 min. The samples were then separated on 10% tris-glycine SDS-PAGE or 16% tris-tricine SDS-PAGE and transferred onto an immobilon-PTM polyvinylidene fluoride membrane. To block nonspecific binding, the membranes were incubated with 5% nonfat milk in Tris-buffered saline containing 0.1% Tween-20 at 37 °C for 1 h. The target proteins were immunoblotted with primary antibody overnight at 4 °C. After incubation with goat anti-rabbit IgG (1:3000; Abcam) at room temperature for 1 h, the protein was detected with the Bio-Rad Imager using ECL Western blotting substrate (Pierce, Waltham, USA).

### Aβ ELISA

Mouse hippocampal homogenates or cell culture media were collected. To prevent Aβ degradation, lysis buffer was added with a protease inhibitor (Roche, Basel, Switzerland). The level of Aβ_40_/Aβ_42_ was determined using an Aβ_40_/Aβ_42_ ELISA Kit (R&D). Samples were measured by a microplate reader (Bio Tek Synergy H1, Winooski, USA) at 450 nm.

### Morris water maze

The Morris water maze test was introduced to detect hippocampal-based spatial memory in mice at the ages of 9 and 12 months, as described previously.^50,51^ The maze consists of a 150-cm diameter circular stainless-steel pool filled with nontoxic white paint, and the temperature was maintained at 24 ± 1 °C. Each mouse performed a 120-s free swim to adapt to the maze 24 h before spatial learning. Then, the mice were trained to search the hidden platform (13 cm in diameter) for four trials per day for 5 consecutive days. Upon failure to reach the hidden platform in 120 s, mice would be guided to the platform where they stayed for 20 s. A retrieval test was conducted 24 h after the last learning trial. The Any-maze tracking system (Stoelting Co., Wood Dale, USA) was used to record escape latency.

### Electrophysiology in vitro

Mice (12 months old) were killed, and hippocampal slices (400-μm thick) were cut coronally with a vibratome (VT1200S, Leica, Wetzlar, Germany) at 95% O_2_ and 5% CO_2_ and then transferred into a submersion-type incubation chamber for a 2-h recovery at 35 °C.^52^ Field excitatory postsynaptic potentials were recorded from hippocampal CA1 stratum radiatum by stimulation of the Schaffer collateral-commissural pathway. Theta burst stimulation was delivered to induce LTP after obtaining a stable baseline. Data acquisition was performed with the PatchMaster v2.73 software (HEKA Electronic, Lambrecht/Pfalz, Germany).

### Transmission electron microscopy

Transmission electron microscopy (TEM) was used to detect the ultrastructures and neurons, as described previously.^53^ Mice were overdosed with urethane and then transcardially perfused with 2.5% glutaraldehyde. Each mouse brain was rapidly separated on ice, and a 1 mm tissue sample was excised from the hippocampal CA1 area. The samples were then fixed in 4% glutaraldehyde for more than 24 h and embedded in Epon812 epoxy resin. Then, the samples were sliced into 1-μm-thick flakes. Philips EM208S TEM (Philips, Amsterdam, Netherlands) was used to observe the ultrastructures and neurons after double staining with uranyl acetate and lead citrate. The presence of at least three vesicles in the presence of the presynaptic bouton and a PSD was used to identify synapses. Random synaptic images obtained according to a previously used method^53,54^ were used to measure the number of synapses and PSD synaptic cleft width and thickness. All measures were carried out in a double-blind manner.

### Luciferase assay

To determine the promoter activity, plasmids containing the promoter regions of the human APP and BACE1 genes were constructed.^3,55^ The plasmids were transfected into N2A^APP^ cells using Lipofectamine 2000 (Invitrogen, Carlsbad, USA), and then MAPK inhibitors were administered for 24 h. The Dual-Luciferase Reporter Assay System (Promega, Madison, USA) was used to measure the activities of firefly luciferase and Renilla luciferase sequentially.

### Aβ oligomer preparation

The Aβ_42_ peptide was obtained from GL Biochem Ltd. (Shanghai, China). To enhance oligomer formation, 1 mm hexafluoroisopropanol was used to dissolve Aβ_42_, which was then evaporated to form a dried film. The film was then dissolved in dimethyl sulfoxide to 5 mm, which was then mixed with PBS and incubated for 48 h at 4 °C. After incubation, the preparation was centrifuged (14,000× *g*, 10 min), and the supernatant was collected.

### Cycloheximide treatment

For the MKP-1 degradation experiment (half-life measurements), Aβ_42_ (10 μm) was used to treat N2A cells for 24 h. For the APP and BACE1 degradation experiments, N2A^APP^ cells were treated with ERK inhibitor and LV_MKP-1_ for 48 h. N2A and N2A^APP^ cells were harvested at different time points after cycloheximide treatment (100 μg/ml).

### EMSA

EMSA was conducted as described previously.^37^ In brief, nuclear extracts of HEK 293 cells were prepared by a cytoplasmic nuclear isolation kit (Invent Biotechnologies, EdenPrairie, USA) supplemented with a protease inhibitor. An IRDye 700–labeled Sp1 oligo (5′-ATTCGATCGGGGCGGGGCGAGC) was mixed with nucleoprotein (2 μg) at room temperature for 30 min in the dark, and the gels were scanned by using the Odyssey system (LI-COR Biosciences, Lincoln, USA). Unlabeled wild-type and mutant (5′-GGTAACTACTAAGTTATTTTCAAGCTACTTAA) Sp1 were used to compete for binding in the competition assay.

### Statistical analysis

All data are expressed as the mean ± SEM. ANOVA or two-tailed Student’s *t* tests were used to analyze the data as appropriate.

## Supplementary information

Supplementary Materials
