# Supplementary material for: MKP-1 reduces Aβ generation and alleviates cognitive impairments in Alzheimer’s disease models
Source: Signal Transduct Target Ther. 2019 Dec 6;4:58. doi: 10.1038/s41392-019-0091-4 (PMC6895219; doi:10.1038/s41392-019-0091-4)
Supplement: Supplementary file 1 — Supplementary Materials [file 41392_2019_91_MOESM1_ESM.docx]

Supplementary Materials for

**MKP-1 reduces Aβ generation and alleviates cognitive impairments in Alzheimer's disease models**

Yehong Du^1^, Yexiang Du^2^, Yun Zhang^3^, Zhilin Huang^1^, Min Fu^1^, Junjie Li^1^, Yayan Pang^1^, Peng Lei^4^, Yu Tian Wang^1,5^, Weihong Song^1,3*^, Guiqiong He^2*^, Zhifang Dong^1*^

^1^Pediatric Research Institute, Ministry of Education Key Laboratory of Child Development and Disorders, National Clinical Research Center for Child Health and Disorders, China International Science and Technology Cooperation Base of Child Development and Critical Disorders, Chongqing Key Laboratory of Translational Medical Research in Cognitive Development and Learning and Memory Disorders, Children’s Hospital of Chongqing Medical University, Chongqing 400014, PR China.

^2^Department of Anatomy, Basic Medical College, Chongqing Medical University, Chongqing 400016, PR China.

^3^Townsend Family Laboratories, Department of Psychiatry, The University of British Columbia, Vancouver, BC V6T 1Z3, Canada.

^4^West China School of Basic Medical Sciences and Forensic Medicine, Sichuan University, Chengdu, 610041, Sichuan, China.

^5^Brain Research Centre, The University of British Columbia, Vancouver, BC V6T 2B5, Canada.

Correspondence to: [zfdong@cqmu.edu.cn](mailto:zfdong@cqmu.edu.cn) (Z.D.), [guiqionghe@hotmail.com](mailto:guiqionghe@hotmail.com) (G.H.) or [weihong@mail.ubc.ca](mailto:weihong@mail.ubc.ca) (W.S.)

**
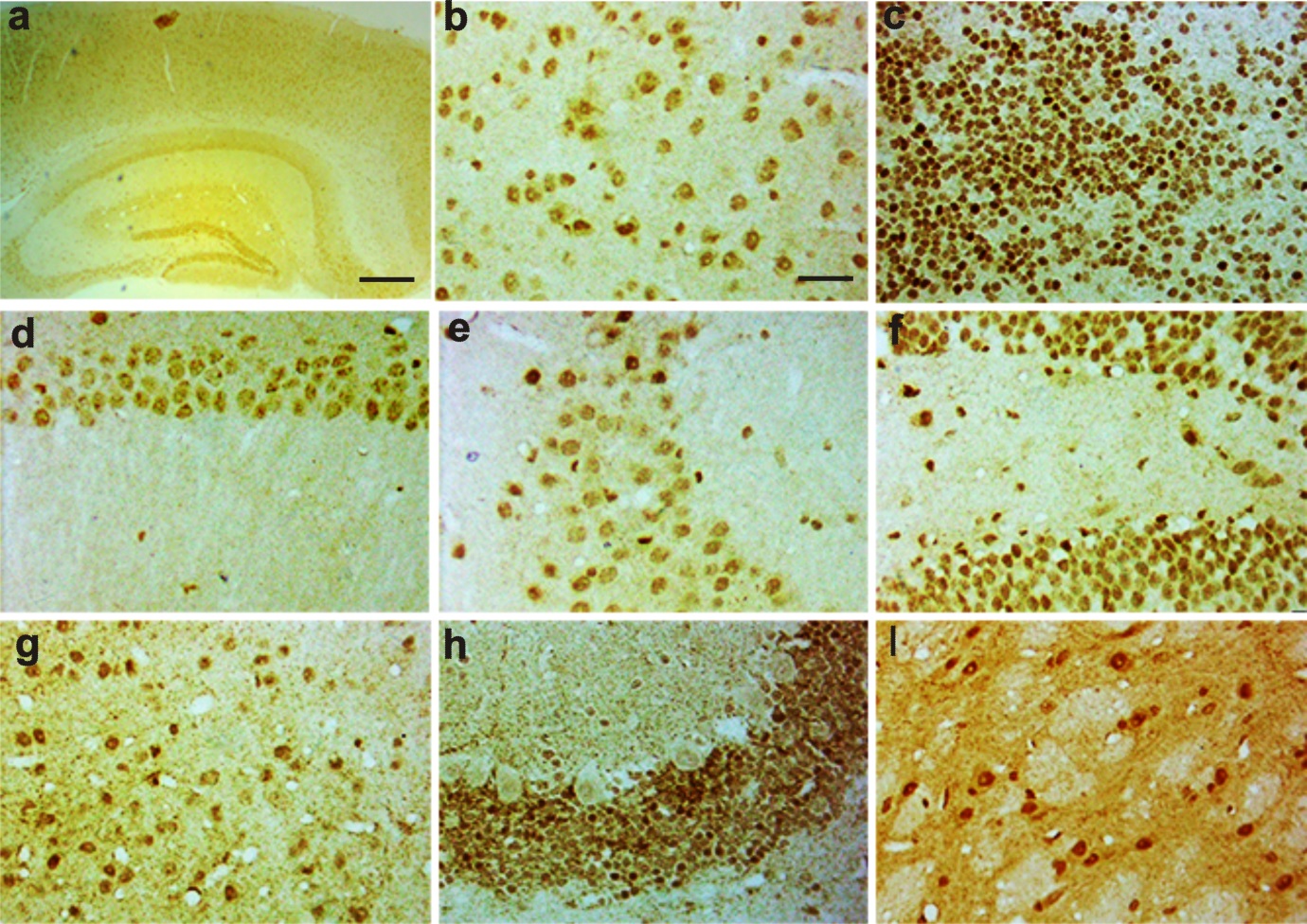
**

**Supplementary Fig. S1. Expression of MKP-1 in the brain of mice.** IHC staining of brain slice (a), cerebral cortex (b), olfactory bulb (c), hippocampal CA1 (d), hippocampal CA3 (e), dentate gyrus (f), thalamus (g), cerebellum (h) and basal forebrain (i). Scale bar: 500 μm for a and 50 μm for b-i.

**
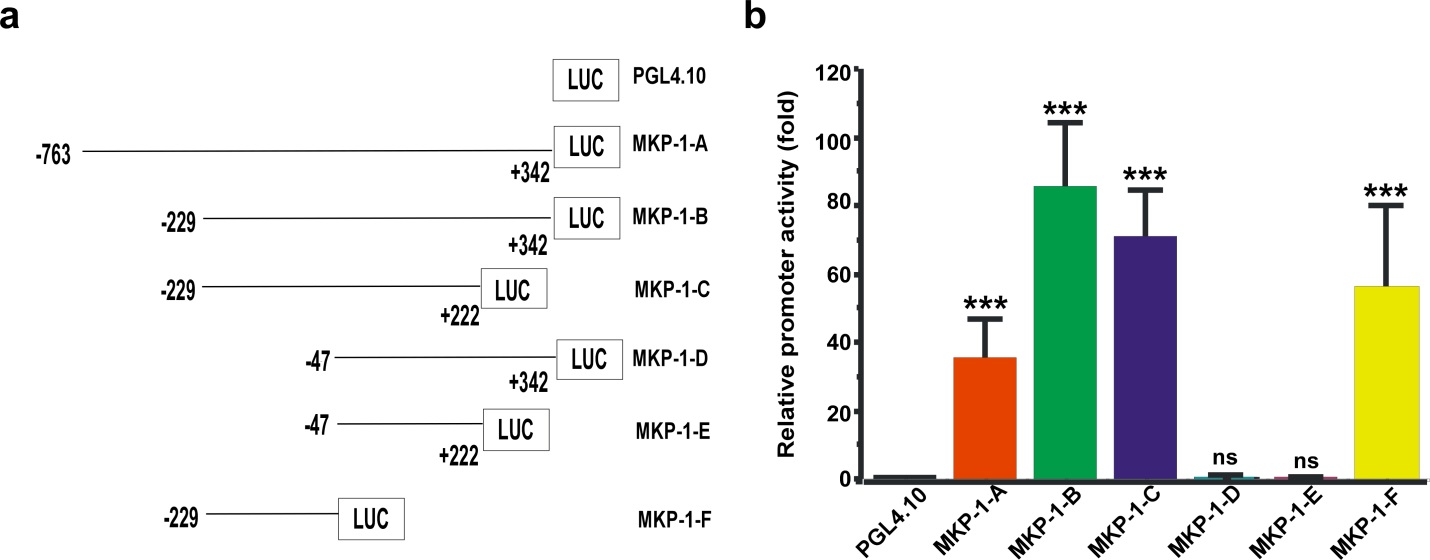
**

**Supplementary Fig. S2. Deletion analysis of the human MKP-1 gene promoter.** (**A**) Schematic diagram of the MKP-1 promoter deletion constructs in pGL4.10-basic vector. (**B**) The deletion plasmids were cotransfected with pRLuc into HEK293 cells. Twenty-four hours after the transfection, the luciferase activity was measured and expressed in relative luciferase units (RLU). **p* < 0.05, ****p* < 0.001 by one-way ANOVA. n = 5 in each group.

**
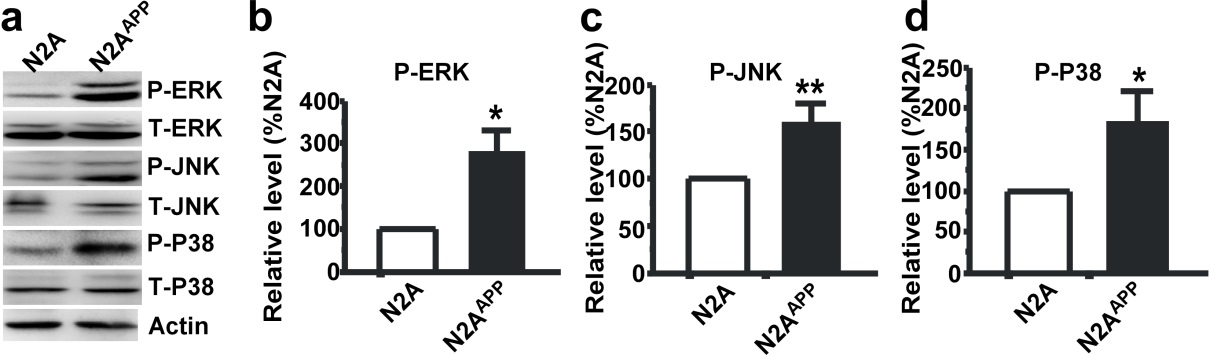
**

**Supplementary Fig. S3. MAPKs are activated in N2A^APP^ cells.** (**a**) Representative immunoblot and quantification of the expression of P-ERK (**b**), P-JNK (**c**) and P-P38 (**d**) in cell lysis of N2A and N2A^APP^ cells. **p* < 0.05,***p* < 0.01, ****p* < 0.001 by unpaired Student’s *t* test. n = 4 to 6 in each group.

**
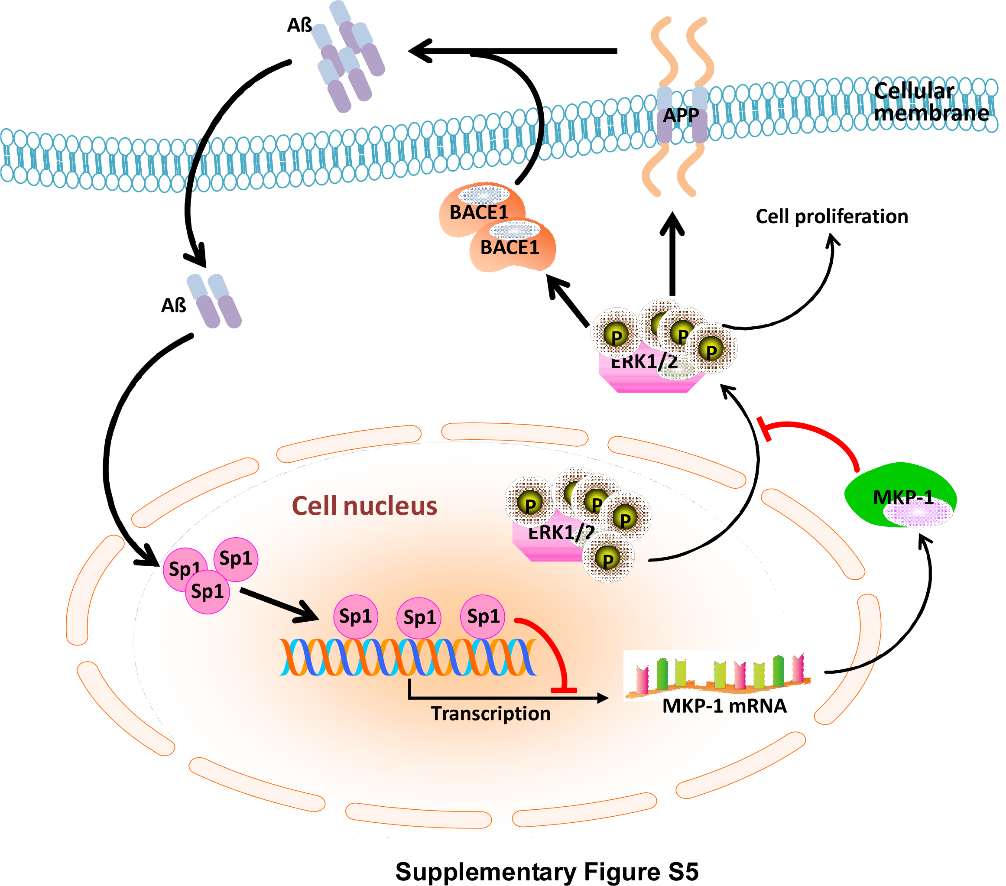
**

**Supplementary Fig. S4. A schematic diagram of potential molecular mechanisms of MKP-1 in AD development.** Aβ increases Sp1 expression, which consequently inhibits MKP-1 transcription. Decreased MKP-1 enhances ERK/MAPK activity through inhibiting its dephosphorylation. Overactivated ERK accelerates Aβ generation through enhancing ERK/MAPK-mediated APP and BACE1 transcriptions.

**Supplementary Table 1. Information for patients with AD and normal controls**

| Case | Sex | Age (years) | Postmortem interval (h) |
| --- | --- | --- | --- |
| AD-1  AD-2  AD-3  AD-4  AD-5  AD-6 | M  M  M  F  F  F | 85  87  73  84  94  60 | 15.5  6.5  9.5  2.3  11.4  7.3 |
| Ctrl-1  Ctrl-2  Ctrl-3  Ctrl-4 | M  M  M  F | 98  87  81  98 | 9.8  17.0  6.1  18.6 |

*Note*: AD = Alzheimer’s disease; Ctrl = normal control.

**Supplementary Table 2. Five potential Sp1-binding sites in the MKP-1 promoter**

| Sp1-1 | TCTCCGCCCCAACTCG |
| --- | --- |
| Sp1-2 | CCCCCACCCCA |
| Sp1-3 | AGCCCTCCTCCTCCCCG |
| Sp1-4 | CCCCCCCTCCCCC |
| Sp1-5 | GGCCCGCCCCGTCCCCC |
